# Adsorptive elimination of Cr(VI) from wastewater using benzalkonium chloride modified Egyptian bentonite

**DOI:** 10.1038/s41598-026-61696-z

**Published:** 2026-07-23

**Authors:** Mariam M. Farouk, Waffa Mekhamer, Olfat M. Sadek, Eslam Syala

**Affiliations:** Department of Materials Science, Institute of Graduate Studies and Researches (IGSR), Alexandria University, 163 Horreya Avenue, Shatby, 21526 Alexandria Egypt

**Keywords:** Organo-clay, Hexavalent chromium, Surface modification, Batch adsorption, Adsorption kinetics, Industrial effluent treatment, Chemistry, Environmental sciences, Materials science

## Abstract

In this study, Egyptian sodium bentonite (NaB) was modified with the cationic surfactant benzalkonium chloride (BAC) to prepare organo-modified bentonite (OMB), which altered the surface chemistry and introduced positively charged adsorption sites, thereby enhancing the adsorption performance. NaB and OMB were characterized by (FTIR), (XRD), (BET), and zero charge point (*pH*_pzc_) measurements. After the modification, the OMB was used to remove highly toxic Cr(VI), commonly found in industrial effluents, from aqueous solutions via batch adsorption. The impacts of BAC loading, pH, exposure time, clay dose, initial Cr(VI) concentration, temperature, and shaking speed were systematically investigated in relation to the sorption process. The optimal conditions for removal were found to be 150% BAC loading (w/w relative to NaB), pH = 2, clay dose = 4 g L^− 1^, 60 min contact time for NaB and 70 min for OMB, 10 mg L^− 1^ initial concentration of Cr (VI) ions, and shaking speed of 200 rpm. Under these conditions, OMB achieved 99.6 ± 0.4% removal ($\:{Q}_{\mathrm{e}}$ ≈ 2.27 ± 0.05 mg g^− 1^), whereas NaB removed only 6.6 ± 0.06% of Cr(VI) ions. Adsorption kinetics followed the pseudo-second-order model, while equilibrium data followed the Langmuir isotherm, indicating surface interactions on the clay surface. Thermodynamic parameters indicated that Cr(VI) adsorption on NaB was endothermic and nonspontaneous, whereas adsorption on OMB was spontaneous and exothermic. These results demonstrate that BAC-modified bentonite is an efficient and low-cost adsorbent for Cr(VI) removal from heavy metal-contaminated water.

## Introduction

Heavy metal contamination caused by rapid industrialization and urbanization poses a serious risk to ecosystems and human health because of the toxicity, persistence, and bioaccumulation of these elements^1^. Heavy metals are naturally deposited in the crust of the Earth, but anthropogenic activities, including industrialization and urbanization, have markedly increased their mobilization and transport in the environment^2^. Among them, chromium is of special concern due to its high toxicity and widespread industrial use^3^. Chromium typically exists as Cr(III) and Cr(VI) forms in water, with Cr(VI) being more mobile, oxidizing, and carcinogenic^3,4^. Major sources of Cr(VI) include chromate pigments, chrome electroplating, ferrochromium smelting, and welding of stainless steels and other alloys containing chromium^3,4^.

Exposure to Cr(VI) can induce DNA damage, nasal mucosa ulceration, emphysema, exaggerated immune responses, acute bronchitis, liver and kidney disorders, and cancers of both the skin and the lungs^4^. Because Cr(VI) is not readily biodegradable, its removal from industrial effluents is essential to protect both human health and the environment^3,4^.

Several physical and chemical approaches can be utilized for Cr(VI) removal, including chemical precipitation, reverse osmosis, ultrafiltration, nanofiltration, coagulation–flocculation, and flotation^3^. However, these methods often suffer from high operational costs, the generation of secondary waste (sludge), and incomplete removal, particularly at low concentrations^3^. Adsorption has emerged as an attractive alternative due to its simplicity, cost-effectiveness, and high efficiency at low contaminant levels^5,6^.

Natural clay minerals, composed of water, alumina, silica, and weathered rocks, are considered promising adsorbents due to their substantial surface area, cation-exchange capacity (Na^+^, Ca^+ 2^, and K^+^), abundance, and low cost^7^. Their overall negative charge, caused by substituting Si^+ 4^ and Al^+ 3^ with other cations, also enhances their sorption capacity. Bentonite, a smectitic clay dominated by montmorillonite, possesses a 2:1 layer structure with net negative charge and exchangeable interlayer cations, making it effective for the adsorption of cationic species^7,8^. However, unmodified bentonite exhibits a limited affinity for anionic contaminants such as Cr(VI) oxyanions. The modification of the surface of clays using organic cations, acids, or thermal treatments can significantly enhance their adsorption capabilities toward specific pollutants^7–9^.

Organo-clays can be obtained by exchanging native inorganic interlayer cations (e.g., Na^+^, Ca^2+^) with organic cationic surfactants, such as quaternary ammonium compounds^7,9^. Benzalkonium chloride (BAC) is a quaternary ammonium surfactant that contains a positively charged head group and a hydrophobic alkyl chain^10,11^. Intercalation of BAC into bentonite can expand the interlayer spacing, modify the surface charge, and introduce new organic functional groups that favor the adsorption of anionic species and organic molecules. The adsorption behavior of heavy metal oxyanions on BAC-modified clays is governed by electrostatic interactions, ion exchange, and complexation processes^10^.

Accordingly, Balighian et al.^12^ utilized modified bentonites treated with Fe(II). The complete elimination efficiency of Cr(VI) was reached at a pH of 2, a 20 mg L^− 1^ initial concentration of Cr(VI), and a dosage of 5 g L^−1^of adsorbent. Hexavalent chromium adsorption was found to occur as a monolayer on the adsorbent surface, since the adsorption data fit satisfactorily with the Langmuir isotherm. Yang et al.^13^ synthesized a chitosan/bentonite composite to remove Cr(VI) from aqueous solutions. The findings indicated that pH = 3 was superior for the high elimination efficiency of Cr(VI). The Redlich-Peterson model best fitted the experimental isotherm data of Cr(VI) adsorption onto the composite, while the kinetic experimental data matched the Elovich model. Consequently, the adsorption process involved multiple layers and generated heat.

Belibağli et al.^14^ synthesized magnetite nanocomposites coated with bentonite as an adsorbent material and examined their effectiveness in Cr(VI) adsorption. Based on the findings, a second- order kinetic model and the Temkin isotherm provided the most accurate description of the adsorption processes. At a concentration of 6.5 mg L^− 1^, the maximum adsorption efficiency for removing Cr(VI) ions was 77.46% at pH 2 and a temperature of 35 °C after 60 min, with an adsorbent concentration of 2.5 g L^− 1^. Velinov et al.^15^ applied wood-aluminum-modified-based sorbents for the removal of Cr(VI) ions and thus the purification of water. The sorbent was prepared by a simple and facile one-stage sonochemical synthesis method. Various characteristic techniques were applied to describe both the sorbents and the whole removal process. The results exhibited that the sorption mechanism was best described by the Langmuir model following pseudo-second-order kinetics, and the maximal sorption capacity of the sonicated sorbent was 104.2 (mg g^− 1^) for Cr(VI) ion removal, which presents a potential material for water treatment and environmental protection.

The present study aimed to prepare BAC-modified Egyptian sodium bentonite (OMB) and evaluate its performance for Cr(VI) removal from solution via the batch adsorption technique. The precise aims of the study are to: (i) synthesize OMB at different BAC loadings and select the optimum loading for Cr(VI) removal; (ii) characterize NaB and OMB by means of FTIR, XRD, BET, and pH_pzc_; (iii) investigate the effects of pH, contact time, clay sorbent dose, starting Cr(VI) concentration, temperature, and shaking speed on Cr adsorption; and (iv) analyze the adsorption kinetics, equilibrium isotherms, and thermodynamic aspects for characterizing the adsorption steps. Although organo-modified bentonites have been previously investigated for Cr(VI) removal, most studies have focused on long-chain surfactants such as CTAB and HDTMA, which often require relatively high adsorbent dosages. Therefore, there remains a need to develop low-cost organoclays capable of achieving high Cr(VI) removal efficiency at low adsorbent doses while providing a comprehensive mechanistic understanding. The novelty of this study lies in: (i) employing BAC as a modifying agent to tailor surface charge and adsorption sites with enhanced electrostatic affinity toward Cr(VI) species, (ii) achieving near-complete removal efficiency (99.6%) at relatively low adsorbent dose compared to previously reported systems, (iii) providing a comprehensive mechanistic interpretation integrating kinetic, isotherm, and thermodynamic analyses regeneration, and real wastewater investigations to provide a reliable evaluation of adsorption performance; and (iv) demonstrating a simple and relatively low-cost modification strategy based on naturally abundant Egyptian bentonite.

Additionally, a comparative evaluation with previously reported organoclays was presented to highlight the relative performance and efficiency of the present BAC-modified system.

Therefore, the principal advantages of the designed OMB system are its high removal efficiency, simple preparation, low-cost raw materials, applicability to real industrial wastewater, and promising regeneration potential. However, limitations such as operation under acidic conditions, which may increase operational costs related to pH adjustment and limit direct large-scale implementation without further optimization, should also be considered.

## Materials, methods, and characterization

### Materials

Egyptian sodium bentonite (NaB) was used as a raw clay adsorbent. Potassium dichromate (K_2_Cr_2_O_7_, (99.9%)) was used as the Cr(VI) stock source, which was purchased from Merck. Hydrochloric acid and sodium hydroxide were obtained from Oxford Laboratory, Mumbai, India. 1,5-Diphenylcarbazide (DPC, 98%) was purchased from Across Organics, USA. Orthophosphoric acid (H_3_PO_4_, 97.99 g mol^− 1^) was obtained from Nano Tech, Egypt. All chemicals were of analytical reagent grade and used without further purification.

### Preparation of Cr(VI) stock solutions

A 1000 mg L^− 1^ Cr(VI) stock solution was prepared by dissolving 2.828 g of K₂Cr₂O₇ in 1 L of distilled water. Working solutions with Cr(VI) concentrations between 10 and 400 mg L^− 1^ were adjusted by appropriate dilution with distilled water and pH adjustment utilizing 0.1–1 M HCl and/or NaOH as required in the range (2–10).

### Preparation of benzalkonium-chloride-modified bentonite (OMB)

Organo-modified bentonite (OMB) was prepared by intercalation of BAC into NaB. Dried NaB (5 g) was dispersed in 50 mL of distilled water. BAC solutions with different loadings (10, 20, 50, 75, 100, 150, and 200% w/w relative to clay) were solubilized in 50 mL of distilled water at the specified concentration and heated at 30 °C for 30 min. The BAC solution was added to the NaB suspension under vigorous stirring, then the mixture was heated at 30 °C for 4 h and then left overnight at room temperature. The solids were separated, dried at 80 °C overnight, ground, and stored in sealed vials. The sample modified with 150% BAC, which provided the highest Cr(VI) removal, was designated OMB and used in subsequent experiments.

### Chromium analysis

The Cr(VI) concentration was determined spectrophotometrically following standard colorimetric procedures using DPC as a complexing reagent. Aliquots of Cr(VI) solution were treated with 500 µL orthophosphoric acid solution and 240 µL DPC solution (in acetone). The absorbance of the purple complex was determined at 540 nm using a UV–Visible spectrophotometer (UV/Visible SP-3000 Plus, Japan), with an appropriate reagent blank as a reference. Calibration curves were constructed at several pH values (2, 4, 6, 8, 10), which followed the Beer–Lambert law over the studied concentration range.

### Characterization of clay adsorbents

The FTIR spectra of NaB and OMB clays were characterized using the standard KBr pellet method (1% w/w sample in KBr) using a Spectrum BX II spectrometer (PerkinElmer, USA) in the range 400–4000 cm^− 1^. The sample compartment was purged with N_2_ between measurements.

XRD crystallographies of both raw and modified bentonites were obtained using a Miniflex 300/600 diffractometer (Rigaku). The basal spacing d(001) was calculated from the position of the first diffraction peak applying Bragg’s law, $\:{d}_{001}=\lambda\:/(2\:\mathrm{s}\mathrm{i}\mathrm{n}{\uptheta\:})$, with Cu Kα radiation (λ = 1.540 Å)^7,16^.

The N_2_ adsorption–desorption isotherms at 298 K room temperature were studied using a fully automated Micromeritics ASAP 2020 surface area analyzer, USA. The BET surface area, pore volume, and pore size distribution were computed from the isotherms according to standard procedures.

**Fig. 1:**
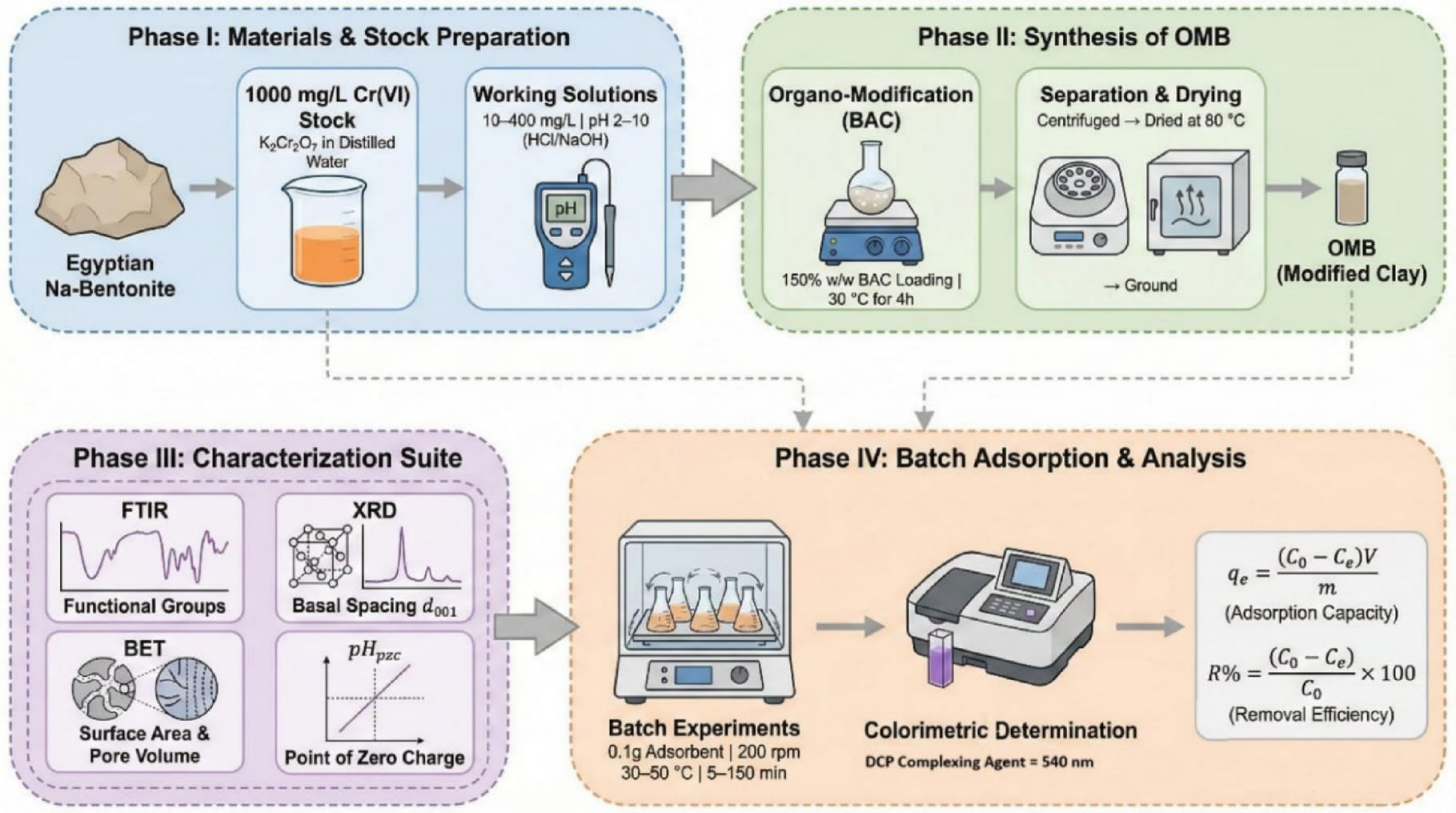
Schematic illustration of the experimental methodology employed in the current study, including the preparation of benzalkonium-chloride-modified bentonite (OMB), batch adsorption experiments for Cr(VI) removal, and the characterization techniques used for evaluating the properties of NaB and OMB sorbents.

The point of zero charge (*pH*_pzc_) was assessed using the pH drift method. For this, 50 mL of 0.01 M NaCl solution was adjusted to initial pH levels ranging from 1 to 10 with 1 M HCl and/or NaOH. Adsorbent (50 mg) was added to the flasks, and after 48 h of equilibration, the final pH was recorded. The difference (*pH*_final_ – *pH*_initial_) was plotted versus *pH*_initial_ (x-axis), and the x-intercept was taken as *pH*_pzc_. A Jenway 3510 pH meter, a UK device, was used to determine the pH. Figure 1 collects the aforementioned experimental procedures (steps) as well as the characterization techniques.

### Batch sorption measurements

Laboratory-scale batch adsorption tests were performed to determine Cr(VI) uptake by NaB and OMB adsorbents under various experimental conditions. In a typical run, a 0.1 g portion of adsorbent was added to 25 mL of Cr(VI) solution in a capped conical flask of specified concentration (10–400 g L^− 1^) and centrifuged at 200 rpm in a temperature-controlled shaker at 30 °C for a predetermined contact time (5–150 min) and pH (2–10). After equilibration, suspensions were filtered, and the concentration of Cr(VI) was measured.

The equilibrium adsorption capacity ($\:{Q}_{\mathrm{e}}$, (mg g^− 1^)) as well as the removal efficiency (%) were calculated by^17^:

1 $$\:{Q}_{\mathrm{e}}=\frac{({C}_{\mathrm{o}}-{C}_{\mathrm{e}})V}{m}$$

2 $$\:R\mathrm{\%}=\frac{\left({C}_{\mathrm{o}}-{C}_{\mathrm{e}}\right)}{{C}_{\mathrm{o}}}\times\:100$$

where $\:{C}_{\mathrm{o}}$ and $\:{C}_{\mathrm{e}}$ are the starting and equilibrium Cr(VI) concentrations (mg L^− 1^), respectively, $\:V$ is the solution’s quantity (L), and $\:m$ is the adsorbent mass (g)^18^.

The influence of solution pH (2, 4, 6, 8, 10) was studied using 25 mL of Cr(VI) initial concentration, 4 g L^− 1^ adsorbent dose, within 120 min of Cr(VI) contact time, 200 rpm shaking speed, at 30 °C room temperature. The pH of the solution was adjusted by adding 0.1 M HCl and/or NaOH and subsequently measured using a laboratory pH meter. 10 mg L^− 1^ initial Cr(VI) concentration was contacted with a sorbent dose of 4 g L^− 1^ at pH = 2 for a period of 5 to 150 min to detect the influence of exposure time on the adsorption process. The solution was continuously agitated using a laboratory shaker at 200 rpm. At predetermined time intervals, 25 mL aliquots were withdrawn using a micropipette and subsequently filtered through a standard filter paper.

0.05 to 0.15 g clay and modified clay adsorbent doses were contacted with 25 mL of Cr(VI) solution at the optimal contact times (60 and 70 min for NaB and OMB, respectively) and shaken at 200 rpm at the background temperature to determine the impact of the clay dose on the removal process.

10–400 mg L^− 1^ initial Cr(VI) concentrations were contacted with 0.1 g of adsorbents and shaken at 200 rpm at 60 and 70 min contact times, for NaB and OMB, respectively, at room temperature to examine the effect of varying the initial concentration of Cr(VI) ions on both the adsorption and removal percentage.

The effect of shaking speed (100, 150, 200 rpm) on Cr(VI) adsorption was determined at 4 g L^− 1^ NaB and OMB dose; at 60 min for NaB and 70 min for OMB contact times, respectively; pH = 2.0; 10 ppm starting concentration; and 30 °C temperature.

The effect of varying the working temperature (30, 40, 50 °C, i.e., 303, 313, and 323 K) on the sorption efficiency was evaluated by mixing 0.1 g of adsorbents at pH = 2.0, 10 g L^− 1^ Cr(VI) concentration, 70 and 60 min for OMB and NaB contact time, respectively, and 200 rpm shaking speed. All batch adsorption experiments were performed independently in triplicate (*n* = 3), and the reported values are presented as the mean ± standard deviation (SD). Error bars in the corresponding figures represent one standard deviation of three independent measurements.

Based on these results, the thermodynamic attributes, namely the free energy change (Δ*G*), enthalpy (Δ*H*), and the entropy (Δ*S*), adjacent to the adsorption process, were mathematically established employing the relations found in reference^5^.

### Kinetic modeling

Adsorption kinetics were studied to explain the mechanism of sorption using the pseudo-first-order, pseudo-second-order, and intraparticle diffusion models.

Pseudo-first-order:

3 $$\:\mathrm{l}\mathrm{n}(qe-qt)=\mathrm{l}\mathrm{n}\:qe-K1t$$

Pseudo-second-order:

4 $$\:\frac{t}{{q}_{t}}=\frac{1}{K2{q}_{e}^{2}}+\frac{t}{{q}_{e}}$$

Intraparticle diffusion:

5 $$\:{q}_{t}={K}_{\mathrm{IPD}}{t}^{1/2}+\mathrm{C}$$

where $\:qt\:$(mg g^− 1^) is the adsorption capacity at time $\:t$ (min), $\:qe\:$is the equilibrium load (mg g^− 1^),

*K*1 (min^− 1^) and *K*2 (g mg^− 1^ min^− 1^) are rate constants, *K*IPD (mg g^− 1^ min^− 1/2^) is the rate constant, and $\:\mathrm{C}$ is the border layer thickness parameter^8,19^.

### Equilibrium adsorption isotherms

Equilibrium data were fitted to Langmuir, Freundlich, Elovich, and Halsey isotherm models^20,21^, to establish the relation between the quantity of sorbate and its concentration, as well as the sorption mechanism.

Langmuir:

6 $$\:qe=\frac{{Q}_{m}{K}_{L}{C}_{e}}{1+{K}_{L}{C}_{e}}$$

Freundlich:

7 $$\:qe={K}_{\mathrm{F}}{C}_{e}^{1/n}$$

Elovich (isotherm form):

8 $$\:\mathrm{l}\mathrm{n}\left(\frac{qe}{{\mathrm{C}}_{e}}\right)=\mathrm{l}\mathrm{n}{\:K}_{\mathrm{E}}-qe/q\mathrm{m}$$

Halsey:

9 $$\:\mathrm{l}\mathrm{n}\:qe=\frac{1}{{n}_{\mathrm{H}}}\mathrm{l}\mathrm{n}\:{C}_{\mathrm{e}}+\mathrm{l}\mathrm{n}\:{K}_{\mathrm{H}}$$

where $\:{Q}_{m}\:$is the monolayer saturation capacity (mg g^− 1^), $\:{K}_{\mathrm{L}}$ (L mg^− 1^), $\:{K}_{\mathrm{F}}$ (mg g^[− 1^ and $\:n$ are the Langmuir and the Freundlich constants, while $\:{K}_{\mathrm{E}}$, $\:q\mathrm{m}$, $\:{K}_{\mathrm{H}}$, $\:{n}_{\mathrm{H}}$ are Elovich and Halsey constants, respectively. The non-linear forms of the models can be found in reference^18^.

## Results and discussion

### Characterization of NaB and OMB sorbent clays

**Fig. 2:**
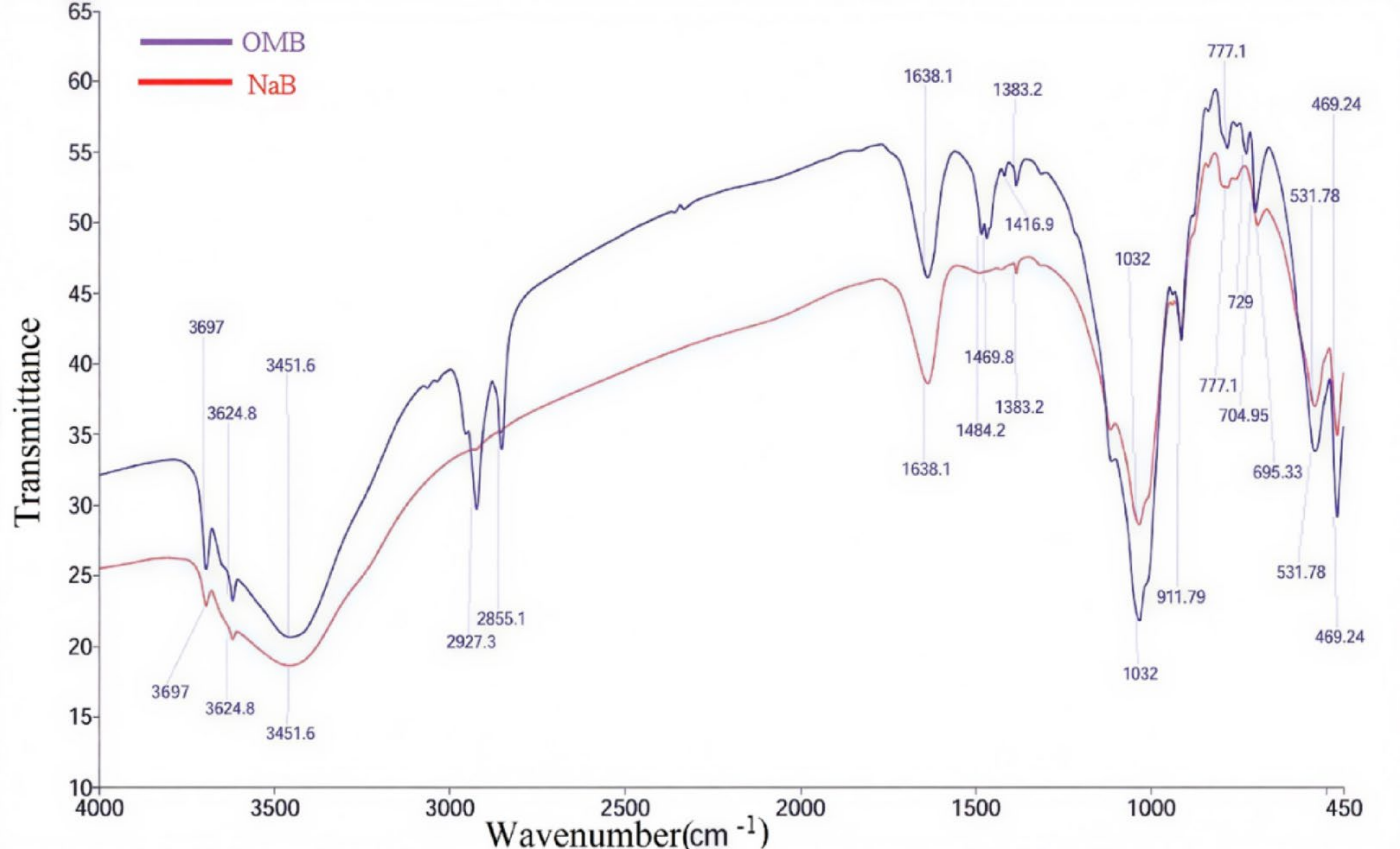
FTIR spectra of NaB and OMB, confirming BAC intercalation into the bentonite structure through the appearance of additional organic vibration bands.

The FTIR spectra of NaB (Fig. 2) showed OH stretching bands at 3697 and 3624 cm^− 1^, corresponding to the Si–OH and Al–OH clusters. The wide band at 3451.6 is assigned to interlayer water, while the peak at 1638.1 cm^− 1^ denotes the bending of the –OH group. An intense band at 1032 cm^− 1^ is due to Si–O stretching in layered silicates. The bands at 531.8 and 469.2 cm^− 1^ stand for Al–O–Si and Si–O–Si bending vibrations^22^. After BAC modification, new bands appeared at 2927.3 and 2855 cm^− 1^, corresponding to the asymmetric and symmetric CH_2_ stretching of the BAC alkyl chains, while bands at 1469.8 and 777 cm^− 1^ accompany the stretching vibration of C = C bonds and the out-of-plane bending vibration of aromatic rings^7,9,13,14,22,23^. These bands confirm the presence of BAC in the OMB spectrum. Additionally, the characteristic NaB bands were retained with slight shifts, verifying the successful intercalation of BAC into the clay galleries.

**Fig. 3:**
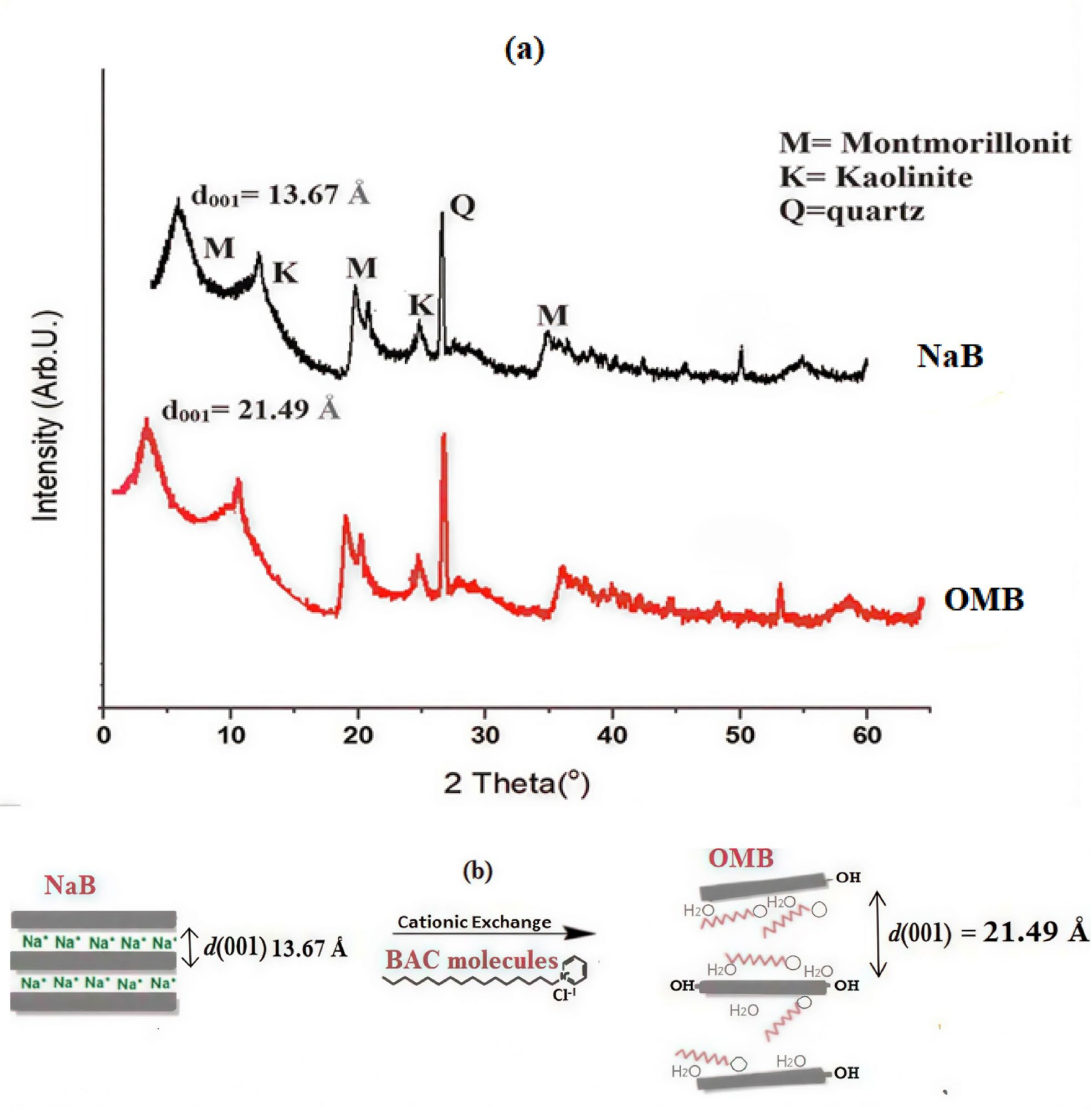
**(a)** X-ray diffraction (XRD) patterns of NaB and OMB sorbents displaying the variation in basal spacing after BAC modification, and (**b**) schematic representation of the intercalation of BAC molecules within the bentonite interlayer galleries, leading to expansion of the clay layer.

Figure 3**(a)** reveals the XRD spectra of NaB and OMB clays, showing that NaB exhibited a basal spacing d_001_ of 13.67 Å corresponding to 2θ = 6.46°, characteristic of clay minerals montmorillonite kaolinite^24^. After modification with BAC, the basal spacing increased to 21.49 Å at 2θ = 4.1° for OMB (as in Fig. 3**(b)**), indicating expansion of the NaB interlayer. The insertion of BAC molecules induced the ion exchange process of sodium cations (Na^+^) located inside the interlayer of sodium bentonite with the quaternary ammonium cation of BAC^24^. This increase in d_001_ confirms the organization and involvement of surfactant chains in the galleries, consistent with the FTIR results.

**Fig. 4:**
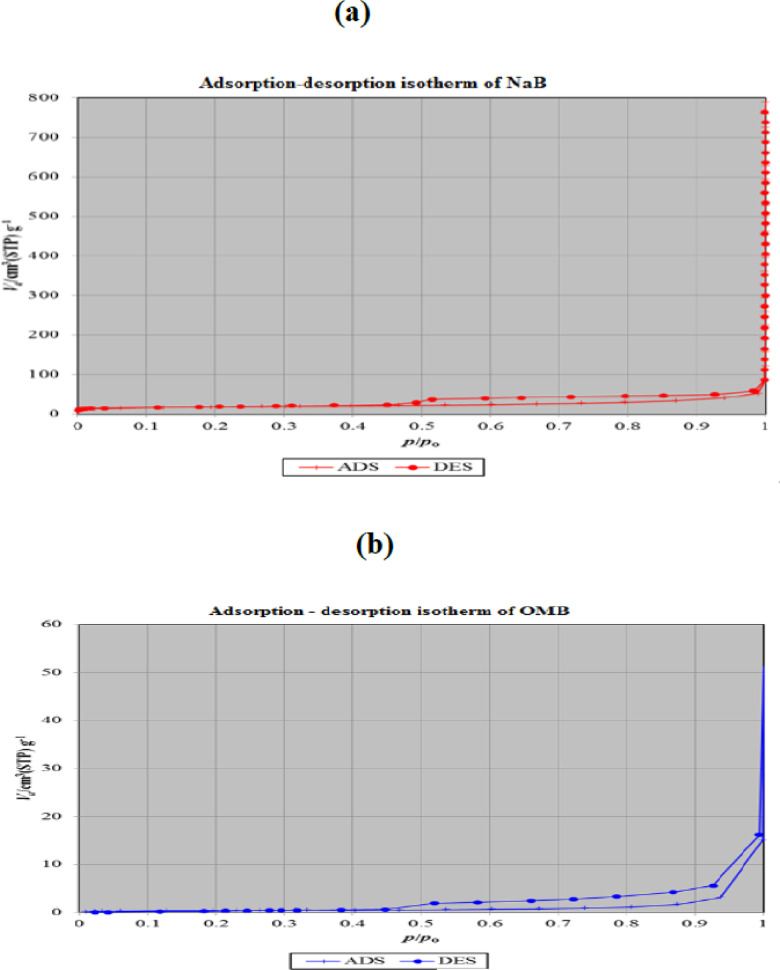
N_2_ adsorption - desorption isotherms for (**a**) NaB and (**b**) OMB sorbents measured at room temperature, illustrating the effect of BAC modification on the textural properties of the adsorbents.

The mesoporous characteristics of NaB and OMB sorbents were fundamentally confirmed from the N₂ adsorption–desorption isotherms and pore size distribution analysis according to the IUPAC classification, which is widely accepted for identifying mesoporous structures within the 2–50 nm range, as seen in Fig. 4^20^. Similar mesoporous characteristics and surface modifications have been reported for organoclay systems in recent adsorption studies^25^.

NaB exhibited a BET surface area of 60.852 m² g^− 1^, a pore volume of 0.0877 cm³ g^− 1^, and an average pore size of 5.756 nm, while OMB revealed 1.496 m² g^− 1^, 0.0205 cm³ g^− 1^, and 54.857 nm, respectively, for the same measurements. Although BAC modification reduced the BET surface area and pore volume, the adsorption performance improved because Cr(VI) uptake was governed primarily by the newly introduced quaternary ammonium functionalities rather than by the total nitrogen-accessible surface area. The surfactant molecules partially occupied pore entrances and interlayer regions, thereby reducing the BET-measured surface area, while simultaneously generating positively charged adsorption sites with strong affinity toward chromate species. Furthermore, the increase in basal spacing observed by XRD suggests that the BAC intercalation changed the clay microenvironment and enhanced the accessibility of functional adsorption sites. Therefore, the adsorption process appears to be controlled mainly by surface chemistry and electrostatic interactions rather than by physical surface area alone. The use of a BAC loading corresponding to 150% of the clay cation-exchange capacity may have promoted the formation of additional surface-associated surfactant layers; however, confirmation would require advanced microscopic characterization.

**Fig. 5:**
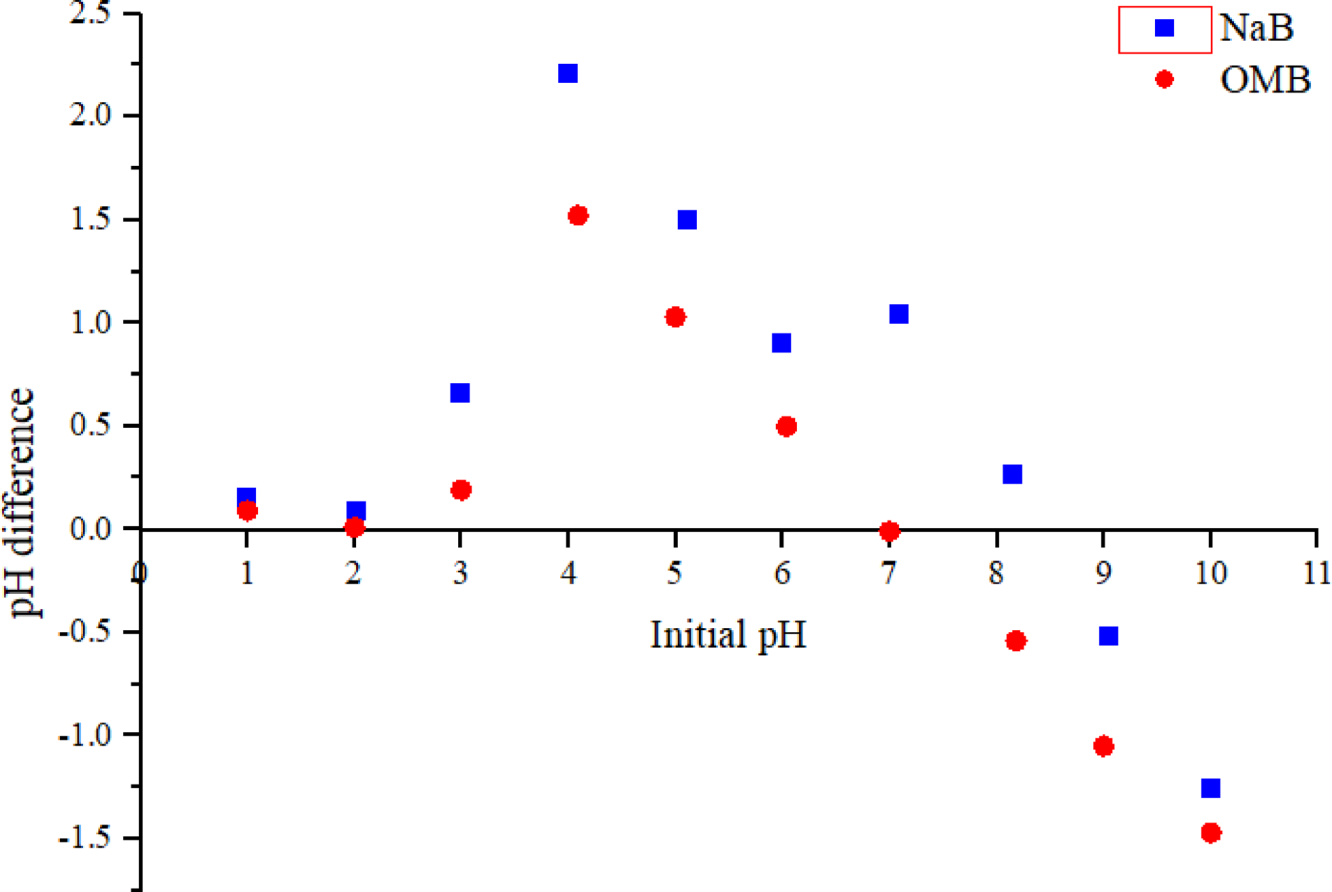
Determining the point of zero charge of NaB and OMB sorbents by pH drift method, illustrating the effect of BAC modification on the surface charge properties of bentonite.

The *pH*_pzc_ values of NaB and OMB determined by the pH drift method were 8.5 and 7.0, respectively, as represented by Fig. 5, indicating that OMB is positively charged below pH 7 and negatively charged above pH 7, which is relevant for the adsorption of anionic Cr(VI) species^26–28^.

### Batch adsorption studies

#### Determining the optimum value and the effect of both BAC loading and shaking speed on the removal percentage of Cr(VI) ions

**Fig. 6:**
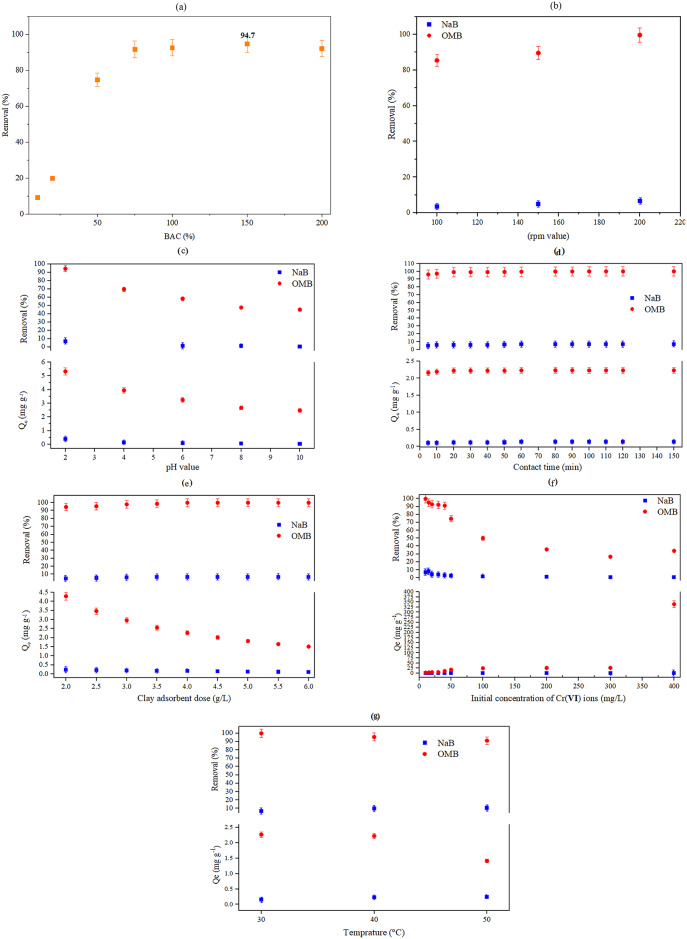
Effect of operational parameters on Cr(VI) adsorption by NaB and OMB sorbents: (**a**) BAC loading, (**b**) shaking speed, (**c**) solution pH, (**d**) contact time, (**e**) adsorbent dose, (**f**) initial Cr(VI) concentration, and (**g**) temperature. The adsorption performance was evaluated in terms of removal efficiency (%) and equilibrium adsorption capacity (*Q*_e_, mg g⁻¹). Error bars represent the standard deviation of triplicate measurements. Experimental conditions were adjusted individually for each parameter while maintaining the remaining adsorption conditions constant.

The role of BAC loading on Cr(VI) removal was evaluated by treating NaB with 10, 20, 50, 75, 100, 150, and 200% BAC (w/w). The Cr(VI) removal efficiency increased markedly with BAC loading up to 75% and continued to increase more gradually up to a maximum value of 150%, then slightly decreased, reaching 200% as represented in Fig. 6. **(a)**. Recent studies (2022–2025) have demonstrated that the growing potential of surfactant-modified clay materials for enhanced anionic contaminant removal is due to the improved electrostatic adsorption and surface accessibility^29–32^.

The slight decrease in Cr(VI) removal at higher BAC loadings may be attributed to the dense packing of the surfactant layers that limits the accessibility of interlayer sites to Cr(VI). Accordingly, the OMB prepared with 150% BAC was selected as the optimal ratio and chosen for subsequent experiments, so this factor was examined.

Related to the variation of the shaking speed from 100 to 200 rpm in subsequent experiments at room temperature, 60 and 70 min adsorption contact times, pH = 2, and a starting concentration of 10 mg L^− 1^ of Cr(VI), it had a modest effect on the sorption process (removal%), as noticed also in Fig. 6. (**b**). Higher shaking speeds slightly increased Cr(VI) removal, particularly for OMB (from 85 to 99%), due to its higher pore size (54.7 nm) compared to 5 nm for NaB (which enhanced only from 3.5 to 6.6%). The increase in adsorption efficiency with rising shaking speed may be ascribed to improved dispersion, mass transfer, and minimized external diffusion resistance. Under appropriate agitation conditions, particle aggregation and stagnant boundary layers are reduced without generating excessive vortex effects, resulting in an enhanced Cr(VI) sorption efficiency^17^. The optimum shaking speed was determined to be 200 rpm.

### pH-dependent behavior of the solution

For both NaB and OMB, Cr(VI) removal and *qe* decreased markedly as the pH increased from 2 to 10. At pH 2, OMB achieved 94.4 ± 0.5% removal ($\:qe\:$≈ 5.32 (mg g⁻¹)), whereas NaB removed only 6.68 ± 0.06% (*qe* ≈ 0.377 (mg g⁻¹)). At higher pH, removal efficiencies were much lower for both adsorbents, as seen in Fig. 6**(c)**.

Generally, Cr(VI) exists in the solution as HCrO^4−^, Cr_2_O_7_^2−^, and CrO_4_^2−^ anions, with HCrO^4−^ dominating at pH 2–4 and CrO_4_^2−^ at pH 9–12^13^.

At pH 2, the OMB surface became positively ionized (pH < *pH*_pzc_) and electrostatically attracted both HCrO_4_− and Cr_2_O_7_^2−^ species found in the solution, thus favoring the adsorption. At higher pH, deprotonation reduces the positive surface charge, while increased OH^−^ competition lowers Cr(VI) uptake. Thus, pH 2 was selected as the optimum value for the adsorption process. Although the highest Cr(VI) removal efficiency was achieved at pH 2, which is consistent with the predominance of protonated adsorbent surfaces and favorable electrostatic attraction of HCrO^4−^ species, the requirement for acidic conditions may limit large-scale practical adsorption implementation. Adjustment of wastewater to low pH values could increase operational costs due to acid consumption and post-treatment neutralization requirements. However, many industrial effluents containing Cr(VI), such as electroplating and metal-finishing wastewater, are already acidic in nature, which may reduce the need for extensive pH adjustment and thus cost. In addition, the BAC-modified bentonite exhibited stable adsorption performance during the experimental contact times under acidic conditions, suggesting a reasonable short-term stability of the organoclay system. However, further studies are recommended to evaluate long-term chemical stability and adsorption performance under near-neutral pH conditions before full-scale application.

### Effect of contact time on the adsorption

The effect of contact time revealed that Cr(VI) removal increased rapidly during the initial adsorption stage, followed by a gradual approach toward equilibrium around 60 min for NaB and 70 min for OMB.

The maximum removals were 6.6 ± 0.06% and 99.6 ± 0.4% for NaB and OMB, respectively, as exhibited in Fig. 6. **(d)**, which reveals that the removal efficiency (%) exhibited a sharp increase during the early adsorption stage, indicating rapid occupation of the available adsorption sites^33,34^. The relatively rapid adsorption behavior may be attributed to the low initial Cr(VI) concentration employed in the kinetic experiments and the high affinity of OMB unoccupied active sites toward Cr(VI) species. At this stage, the larger average pore size of OMB (54.75 nm) compared with NaB (5 nm), as stated by the BET surface area result, may have facilitated mass transfer and improved accessibility of the adsorption sites for Cr(VI) species, in addition to between the OMB layers. This stage was followed by a slower phase, in which sites became occupied and the diffusion into pores became rate-limiting.

### The influence of the clay adsorbent dose

Increasing the adsorbent quantity from 2 to 4 g L^− 1^ increased Cr removal from 4.7 ± 0.05% to 6.7 ± 0.06% with NaB and from 94.3 ± 0.5% to 99.6 ± 0.4% with OMB while conducting the experiments at 10 mg L^− 1^ Cr(VI) fixed concentration, pH = 2, shaking speed: 200 rpm, 30 °C room temperature, as exhibited in Fig. 6. **(e)**. This behavior can be ascribed to the increased availability of ion-exchange and adsorption sites as the adsorbent dose increases^35^. Above 4 g L^− 1^ (up to 6 g L^− 1^ in the present case), the removal efficiency did not significantly increase, indicating the saturation of the active sites^36^. The adsorption capacity $\:{Q}_{\mathrm{e}}\:$(mg g⁻¹) decreased from 0.21 to 0.1 (mg g⁻¹) in the case of NaB and from 4.28 to 1.51 (mg g⁻¹) in the case of OMB with increasing the adsorbent dose, due to particle crowding and underutilization of adsorption sites (solid concentration effect). Therefore, an adsorbent dose of 4 g L^− 1^ was selected as the optimum operating condition.

### Assessment of adsorption process at different initial Cr(VI) concentrations

As the initial Cr(VI) concentration increased from 10 to 400 mg L^− 1^, the removal% of Cr(VI) decreased from 6.68 to 0.459%, while the *qe* increased from 0.157 to 0.455 mg g^− 1^ for the NaB adsorbent. Similarly, for OMB clay, the removal percentage decreased from 99.6 to 26.46%, while $\:{Q}_{\mathrm{e}}$ increased from 2.27 to 26.24 mg g^− 1^, approaching a plateau beyond ~ 200 mg L^− 1^ for both sorbents, as seen in Fig. 6 (**f**). This phenomenon arises from the higher concentration of Cr(VI) ions in comparison to the active sorption sites at higher concentrations, leading to a lower removal percentage. On the other hand, higher adsorption capacity was due to increased driving force, diffusion of Cr(VI), and a stronger concentration gradient^37^.

### Effect of temperature

For NaB clay, the adsorption magnitude $\:{Q}_{\mathrm{e}}$ increased with rising temperature from 30 to 50 °C at all initial concentrations, as observed in Fig. 6. (**g**). This behavior can be attributed to the escalation in the kinetic energy of Cr ions, which increased collisions between the NaB active site and Cr ions, indicating an endothermic process^38^. In contrast, for OMB clay, *qe* decreased slightly with increasing temperature in the same range, i.e., from 30 to 50 °C, indicating an exothermic adsorption process, which is consistent with the thermodynamic analysis, as will be discussed later, where higher temperature disfavors binding of Cr(VI) to OMB active sites^39^.

### Kinetic modeling

**Fig. 7:**
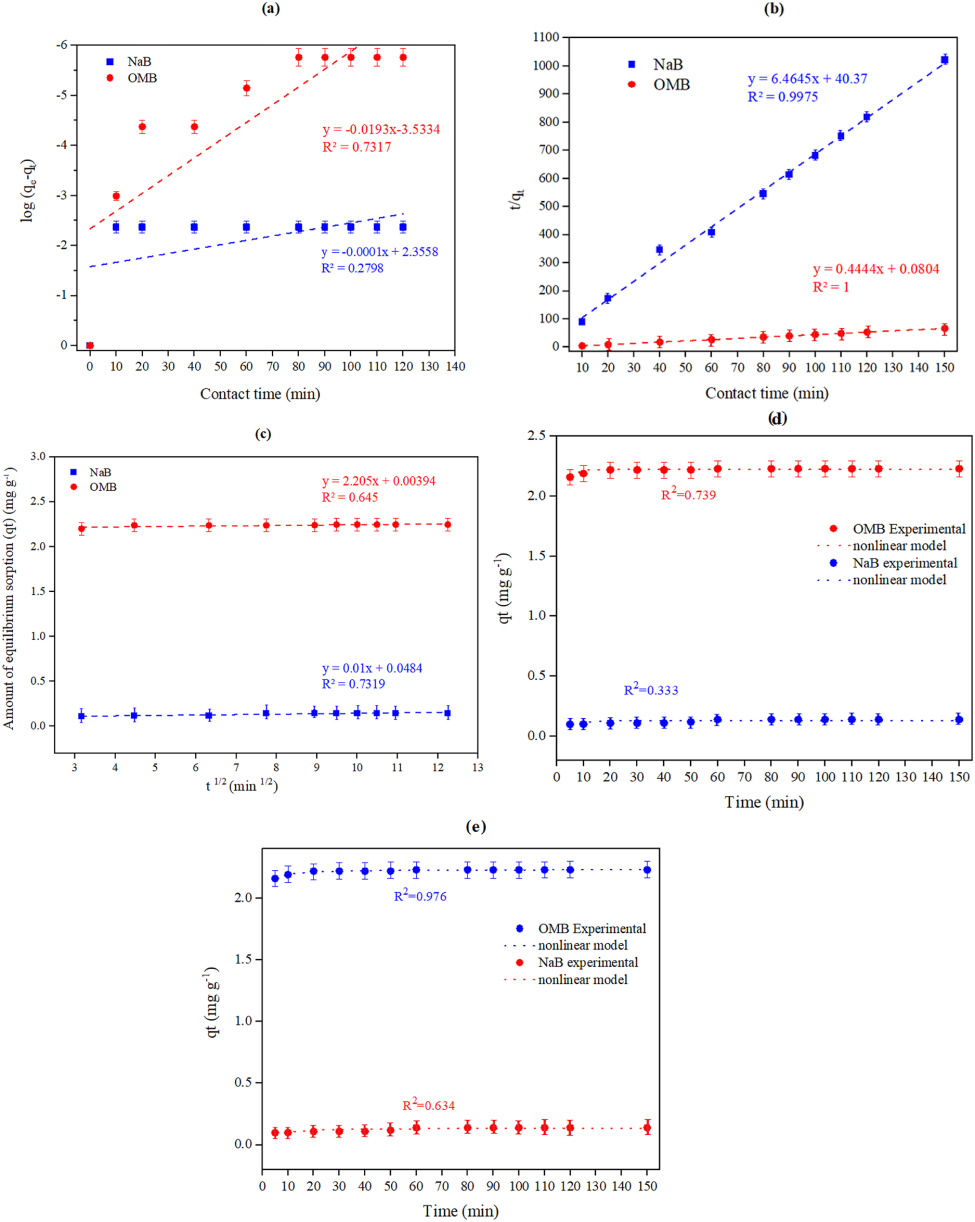
Fitting plots of the kinetic adsorption models for Cr(VI) sorption onto NaB and OMB sorbents: (**a**) linear pseudo-first-order model, (**b**) linear pseudo-second-order model, (**c**) linear intraparticle diffusion model, (**d**) non-linear pseudo-first-order model and (**e**) non-linear pseudo-second-order model. The experiments were conducted at pH 2, 30 °C, 10 mg L⁻¹ initial Cr(VI) concentration, 4 g L⁻¹ adsorbent dose, and 200 rpm shaking speed.

Kinetic data for NaB and OMB at 30 °C room temperature, 10 mg L^− 1^ Cr(VI) initial concentration, pH = 2, 4 g L^− 1^ sorbent dose, 60 and 70 min contact times for NaB and OMB, respectively, and 200 rpm shaking speed were fitted to pseudo-first-order, pseudo-second-order, and intraparticle diffusion models. For NaB, $\:qe$ (exp) ≈ 0.15 ± 0.01 (mg g⁻¹) and pseudo-second-order *qe* (cal) ≈ 0.15 ± 0.01 (mg g⁻¹), with *K*2 ≈ 1.10 ± 0.05 (g mg^− 1^ min^− 1^) and *R*² = 0.9957, as tabulated in Table 1. For OMB, $\:qe$ (exp) ≈ 2.25 ± 0.03 (mg g⁻¹) and pseudo-second-order *qe* (cal) ≈ 2.25 ± 0.03 (mg g⁻¹), with *K*2 ≈ 2.457 ± 0.08 (g mg^− 1^ min^− 1^) and *R*² = 1.000. In contrast, pseudo-first-order fits yielded lower *R*² values, and $\:qe$ (cal) deviated substantially from $\:qe$ (exp), as observed in Fig. 7**(a** and **b)** and Table 1. The poor fitting of the model and the deviation between experimental and calculated adsorption capacities indicate that this model inadequately describes the adsorption behavior. These findings show that the pseudo-second-order (PSO) model best describes the adsorption kinetics, implying that the process is consistent with specific interactions, including electrostatic attraction and/or possible adsorption-related interactions^23,40^. It provided *qe* (cal) similar to $\:qe$ (exp), suggesting better applicability of the model for describing Cr(VI) adsorption onto OMB.

**Table 1:** Kinetic models parameters describing the adsorption of Cr (VI) onto NaB and OMB sorbents.

Models	NaB				OMB			
Pseudo-first- order	*qe* (exp) (mg g⁻¹) 0.15 ± 0.01	*qe* (cal) (mg g⁻¹) 0.095 ± 0.01	*K*1 (min-1) 0.0001 ± 0.00001	*R*2 0.2798	*qe* (exp) 2.25 ± 0.03	*qe* (cal) (mg g⁻¹) 0.029 ± 0.003	*K*1 (min^− 1^) 0.0193 ± 0.001	*R*2 0.7317
Pseudo-second- order		*qe* (cal) (mg g⁻¹) 0.15 ± 0.01	*K*2 (g mg^−1^min^− 1^) 1.1009 ± 0.05	*R* ^2^ 0.9957		*qe* (cal) (mg g⁻¹) 2.25 ± 0.03	*K*2 (g mg^−1^min^− 1^) 2.457 ± 0.08	*R* ^2^ 1
Intraparticle diffusion		*K*IPD (mg g^−1^min^1/2^) 0.01 ± 0.001	C 0.0484 ± 0.003	*R* ^2^ 0.7319		*K*IPD (mg g^−1^ min^1/2^) 0.0874 ± 0.004	C 1.426 ± 0.05	*R* ^2^ 0.645

The model also presented the best kinetic fit (*R*² = 0.9957 for NaB; *R*² = 1.000 for OMB), indicating that the rate-limiting step may involve specific surface interactions or shared/exchanged electron-pair formation between adsorbent (functionalized OMB surface) and adsorbate (Cr(VI) species). The predominance of PSO kinetics observed in this work is consistent with recent Cr(VI) adsorption from aqueous solutions studies^41^.

However, it is important to note that PSO fitting alone cannot definitively distinguish chemisorption from physisorption with strong specific interactions.

The intraparticle diffusion plot ($\:q\mathrm{t}$ vs. t^1/2^ (Fig. 7. **c)**) did not run through the origin for both sorbents, besides its low *R*^2^values, implying that intraparticle diffusion is involved (contributed) to the adsorption process but is not the sole rate-controlling mechanism; boundary layer effect and surface adsorption contribute as well^40^, significantly in Cr(VI) uptake. Furthermore, the mesoporous structure confirmed from BET/BJH characterization likely facilitated Cr(VI) ions movement toward internal adsorption sites by reducing pore diffusion resistance and improving accessibility of active adsorption regions. According to the Boyd diffusion concept, the adsorption mechanism appears to involve combined film diffusion and intraparticle diffusion contributions rather than exclusive pore diffusion control. The higher *K*2 and $\:qe$ for OMB compared with NaB demonstrate the beneficial effect of BAC modification on both adsorption rate and capacity. The present kinetic analysis was performed under representative adsorption conditions to comparatively evaluate NaB and OMB adsorption behavior. Further kinetic investigations at multiple initial Cr(VI) concentrations may provide additional insight into concentration-dependent adsorption mechanisms. However, utilizing the nonlinear forms of the applied linear models on the kinetic data resulted in a satisfying fit of the behavior and confirmed the applied linear models’ results, as presented in Fig. 7**(d** and **e)**.

### Equilibrium isotherms

**Fig. 8:**
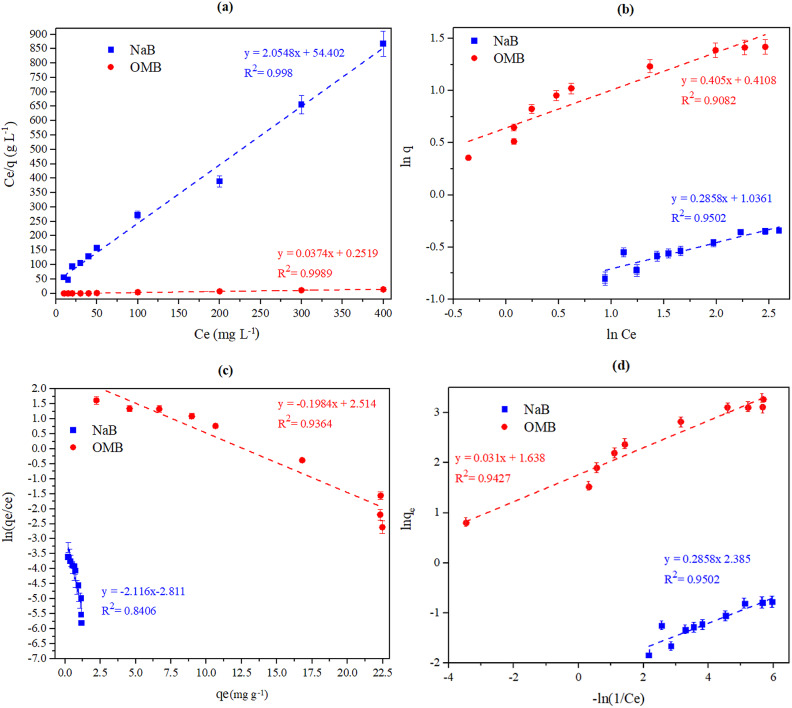
Linear fitting plots of equilibrium adsorption isotherm models for Cr(VI) adsorption onto NaB and OMB sorbents: (**a**) Langmuir, (**b**) Freundlich, (**c**) Elovich, and (**d**) Halsey isotherm models. The plots shown correspond to adsorption equilibrium data obtained at 303 K, while similar analyses were performed at additional temperatures.

Equilibrium Cr(VI) adsorption isotherms for NaB and OMB at 30, 40, and 50 ° C showed L-type behavior according to Giles classification, indicating a strong adsorbate–adsorbent affinity. Langmuir plot ($\:\frac{q\mathrm{e}}{{C}_{\mathrm{e}}}\:$vs. $\:{C}_{\mathrm{e}}$) was linear with a high correlation coefficient (*R*² ≈ 0.996–0.999) for both adsorbents at all temperatures, as observed in Fig. 8**(a)**, where the Langmuir model provided the appropriate fit among those tested. The good applicability of the model suggests a relatively homogeneous distribution of active adsorption sites and predominant monolayer adsorption behavior of Cr(VI) species on the OMB surface, which provided maximum capacities ($\:{Q}_{\mathrm{m}}$) of 26.74 ± 0.65, 22.88 ± 0.58, and 20.79 ± 0.52 (mg g⁻¹) at 303, 313, and 323 K, respectively, with *K*_L_ values between 0.088 and 0.197 L mg^[− 1^, as in Table 2. For NaB, $\:{Q}_{\mathrm{m}}$ values were much lower (≈ 0.49–1.35 (mg g⁻¹)) compared to OMB. The enhanced Langmuir adsorption capacity observed for OMB compared with NaB may be attributed to BAC intercalation, which increased the availability of positively charged adsorption sites and strengthened electrostatic attraction toward anionic Cr(VI) species. The dimensionless separation factor *R*_L_ values were 0 < *R*_L_< 1 over the examined concentration range, reflecting efficient adsorption behavior^19^.

**Table 2:** Parameters of various linear adsorption isotherm models for Cr (VI) on NaB and OMB sorbents.

Model	Parameter	Sorbent	Temperature (K)		
			303	313	323
Langmuir	*qe* (mg g⁻¹)	NaB	0.439 ± 0.02	1.149 ± 0.04	1.193 ± 0.05
		OMB	24.36 ± 0.63	22.35 ± 0.56	19.71 ± 0.50
	$\:{{q}_{\mathrm{c}\mathrm{a}\mathrm{l}}/Q}_{m}$ (mg g⁻¹)	NaB	0.4866 ± 0.02	1.284 ± 0.05	1.350 ± 0.06
		OMB	26.737 ± 0.65	22.883 ± 0.58	20.79 ± 0.52
	*K*_L_ (L mg⁻¹)	NaB	0.0377 ± 0.002	0.02998 ± 0.001	0.03335 ± 0.002
		OMB	0.148 ± 0.006	0.197 ± 0.008	0.088 ± 0.004
	*R*²	NaB	0.998	0.998	0.9958
		OMB	0.9989	0.9998	0.9989
Freundlich	*K*_F_	NaB	0.09 ± 0.01	0.134 ± 0.01	1.092 ± 0.04
		OMB	5.1499 ± 0.12	4.7874 ± 0.11	2.5751 ± 0.08
	1/n	NaB	0.2858 ± 0.01	0.4016 ± 0.02	2.1027 ± 0.07
		OMB	0.31 ± 0.01	0.3173 ± 0.01	0.405 ± 0.02
	*R*²	NaB	0.95	0.88	0.84
		OMB	0.90	0.88	0.90
Elovich	$\:{\mathrm{q}}_{\mathrm{m}}$ (mg g⁻¹)	NaB	0.1228 ± 0.01	0.4725 ± 0.02	0.554 ± 0.03
		OMB	5.3219 ± 0.14	5.040 ± 0.12	5.8685 ± 0.15
	*K*_E_	NaB	1.91 × 10⁻¹⁰ ± 0.08 × 10⁻¹⁰	2.607 × 10⁻³ ± 0.11 × 10⁻³	5.91 × 10⁻³ ± 0.24 × 10⁻³
		OMB	1.967 ± 0.06	1.6476 ± 0.05	1.2184 ± 0.04
	*R*²	NaB	0.84	0.84	0.7185
		OMB	0.93	0.94	0.94
Halsey	$\:{n}_{\mathrm{H}}$	NaB	−3.49 ± 0.12	−2.49 ± 0.09	−2.49 ± 0.08
		OMB	−3.23 ± 0.10	−3.152 ± 0.10	−2.47 ± 0.08
	*K*_H_	NaB	413.61 ± 11.5	149.9 ± 4.8	121.51 ± 4.2
		OMB	197.7 ± 6.3	139.1 ± 4.7	10.32 ± 0.42
	*R*²	NaB	0.9502	0.8823	0.846
		OMB	0.94	0.88	0.91

Regarding Freundlich, Elovich, and Halsey models, they exhibited lower and less consistent *R*² values, particularly for NaB, as tabulated in Table 2 and seen in Fig. 8 (b, c, and d), suggesting that monolayer adsorption at relatively homogeneous sites dominates the Cr(VI) ions uptake under the conditions studied. Specifically, Freundlich model parameters indicated the presence of surface heterogeneity, but the lower fitting correlation compared with Langmuir suggests that multilayer adsorption was less dominant under the investigated conditions.

**Fig. 9:**
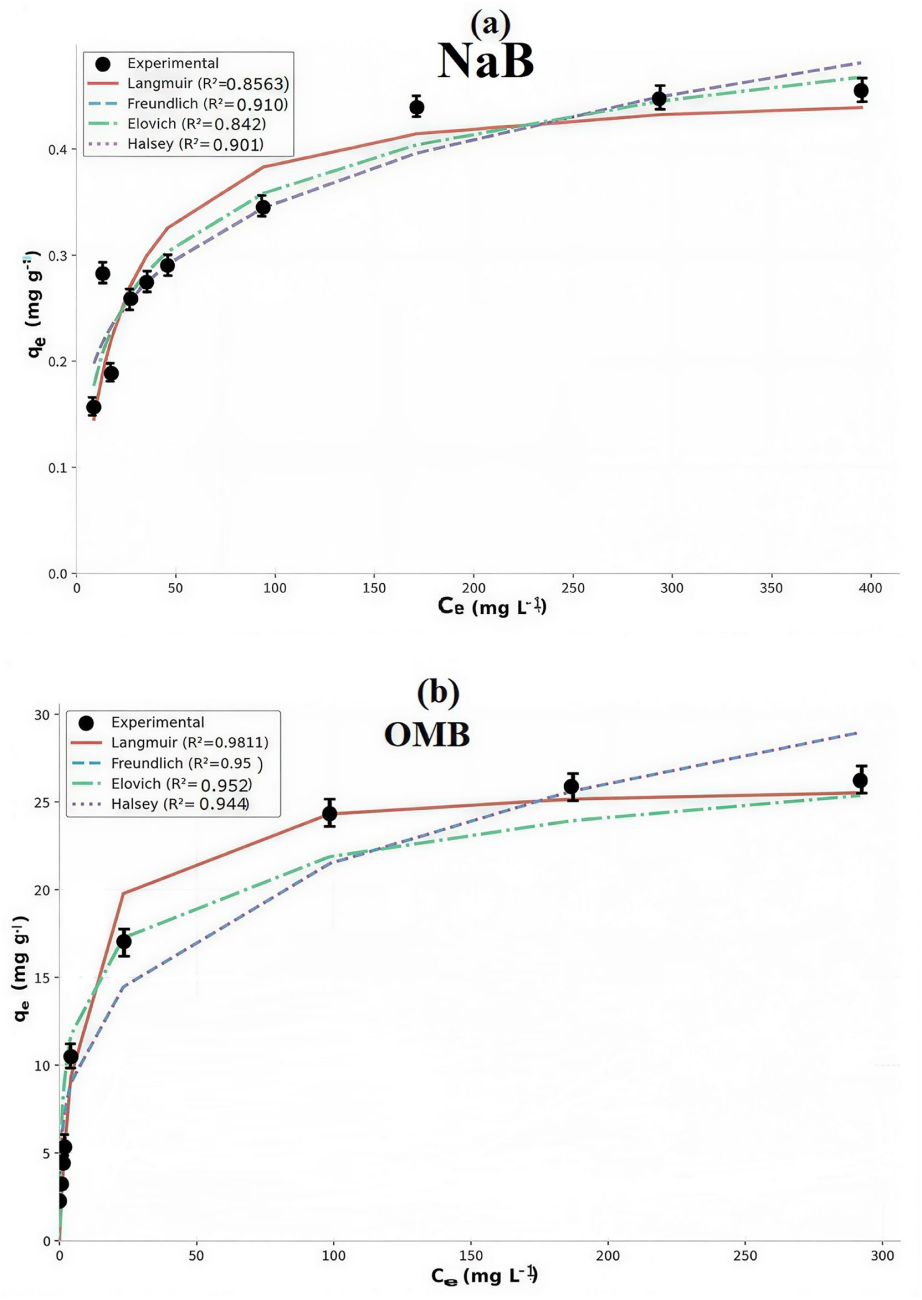
Non-linear fitting plots of adsorption isotherm models for Cr(VI) adsorption onto (**a**) NaB and (**b**) OMB sorbents at different temperatures, applying Langmuir, Freundlich, Elovich, and Halsey isotherm models. The plots shown correspond to adsorption equilibrium data obtained at 303 K (as representative of the studied temperatures).

The high $\:{Q}_{\mathrm{m}}$ of the OMB can be compared favorably with biosorbents and biochars reported in the literature for Cr(VI) removal, such as modified pomegranate peel, rice straw biochar, and plant-based adsorbents^13,37,38,42,43^. Comparison between experimental adsorption capacities and the calculated values obtained from both linear and non-linear Langmuir models revealed that the non-linear fitting generally produced adsorption capacities closer to the experimentally observed *qe* values, particularly for the OMB sorbent. In addition, the non-linear fitting exhibited comparable or improved correlation coefficients (*R*²) relative to the linearized model. These findings indicate that nonlinear regression more accurately described the adsorption equilibrium because it avoided errors introduced by linearization of the isotherm equations, as in Table 3; Fig. 9**(a** and **b)**.

**Table 3:** Non-linear adsorption isotherm parameters applying various models for Cr (VI) uptake on NaB and OMB sorbents.

Model	Parameter	Sorbent	Temperature (K)		
			303	313	323
Langmuir	*qe* (exp) (mg g⁻¹)	NaB	0.439 ± 0.02	1.149 ± 0.04	1.193 ± 0.05
		OMB	24.36 ± 0.63	22.35 ± 0.56	19.71 ± 0.50
	$\:{Q}_{max}$ (mg g⁻¹)	NaB	0.4602 ± 0.02	1.3165 ± 0.05	1.4067 ± 0.06
		OMB	26.1939 ± 0.65	22.5620 ± 0.58	20.4988 ± 0.52
	*K*_L_ (L mg⁻¹)	NaB	0.0416 ± 0.02	0.0283 ± 0.05	0.0307 ± 0.06
		OMB	0.1626 ± 0.65	0.1889 ± 0.58	0.0957 ± 0.52
	*R*²	NaB	0.8563	0.9905	0.9601
		OMB	0.9811	0.9886	0.9898
Freundlich	*K*_F_	NaB	0.0965 ± 0.01	0.1540 ± 0.01	1.2360 ± 0.04
		OMB	5.8424 ± 0.12	5.0957 ± 0.11	2.8644 ± 0.08
	1/n	NaB	0.3293 ± 0.01	0.4302 ± 0.01	1.9916 ± 0.04
		OMB	0.3054 ± 0.12	0.3178 ± 0.11	0.3846 ± 0.08
	*R*²	NaB	0.9105	0.8598	0.8231
		OMB	0.9474	0.9307	0.9456
Elovich	$\:{\mathrm{q}}_{\mathrm{m}}$ (mg g⁻¹)	NaB	0.1287 ± 0.01	0.5011 ± 0.02	0.5908 ± 0.03
		OMB	5.5644 ± 0.14	5.2488 ± 0.12	6.0149 ± 0.15
	*K*_E_	NaB	1.83 × 10⁻¹⁰ ± 0.09 × 10⁻¹⁰	0.0030 ± 0.02	0.0062 ± 0.03
		OMB	1.8428 ± 0.14	1.5938 ± 0.12	1.2714 ± 0.15
	*R*²	NaB	0.8427	0.8385	0.7316
		OMB	0.9522	0.9584	0.9495
Halsey	$\:{n}_{\mathrm{H}}$	NaB	−3.2185 ± 0.12	−2.3245 ± 0.09	−2.1088 ± 0.08
		OMB	−3.1044 ± 0.10	−3.0021 ± 0.10	−2.4016 ± 0.08
	*K*_H_	NaB	398.5221 ± 0.12	158.4402 ± 0.09	132.5801 ± 0.08
		OMB	188.4407 ± 0.10	145.6074 ± 0.10	12.4018 ± 0.08
	*R*²	NaB	0.9017	0.8521	0.8128
		OMB	0.9440	0.9264	0.9367

### Thermodynamic parameters

**Fig. 10:**
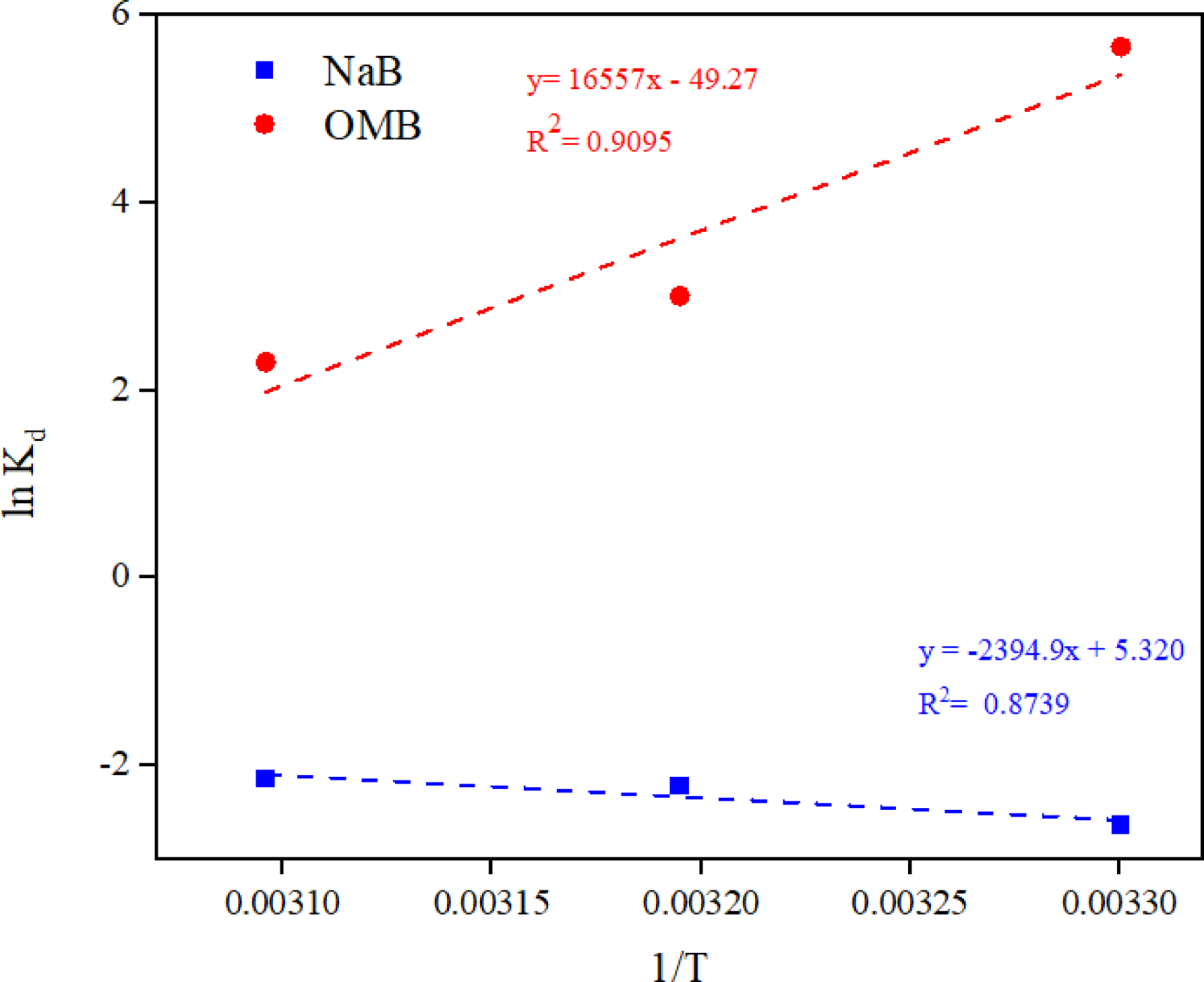
Van’t Hoff plots for determining the thermodynamic parameters of Cr(VI) adsorption onto NaB and OMB sorbents, including Gibbs free energy (Δ*G*), enthalpy (Δ*H*), and entropy (Δ*S*), over the studied temperature range.

Thermodynamic parameters for Cr(VI) adsorption on NaB and OMB were estimated using the Van’t Hoff equation and plot (Fig. 10). The adsorption distribution coefficient (K*d*), defined as the ratio of the equilibrium adsorption capacity ($\:{q}_{e}$) to the equilibrium concentration of Cr(VI) in solution ($\:{C}_{\mathrm{e}}$), was employed for the thermodynamic calculations. Since K*d* obtained from adsorption experiments is not intrinsically dimensionless, it was considered in a normalized (dimensionless) form for application of the Van’t Hoff equation, following the commonly adopted approximation used in adsorption thermodynamic studies^44^. Accordingly, the calculated thermodynamic parameters should be interpreted as approximate indicators of the relative thermodynamic behavior of the adsorption system rather than absolute thermodynamic constants.

For NaB, Δ*G* values were positive (6.64, 5.79, and 5.77 kJ mol⁻¹ at 303, 313, and 323 K), indicating non-spontaneous adsorption of Cr(VI) ions in the studied temperature range. The positive Δ*H* (19.91 ± 0.72 kJ mol^− 1^) and positive Δ*S* (44.23 ± 1.15 J mol⁻¹ K⁻¹) suggested an endothermic process accompanied by increased randomness at the solid–solution interface.

For OMB, Δ*G* values were negative (− 14.27 ± 0.43, − 7.82 ± 0.26, and − 6.18 ± 0.22 kJ mol⁻¹ at 303, 313, and 323 K), demonstrating a feasible and spontaneous adsorption process. The strongly negative Δ*H* (− 137.65 ± 4.8 kJ mol⁻¹) indicates an exothermic adsorption process. While this value exceeds typical magnitudes for purely physical adsorption (− 20 to − 40 kJ mol⁻¹), it is suggested to be consistent with the operation of multiple simultaneous interaction mechanisms, which involve: (i) strong electrostatic attraction between protonated quaternary ammonium groups of BAC and Cr(VI) oxyanions, (ii) other possible surface-mediated interactions involving Cr(VI) species, although the specific nature of these interactions cannot be identified from the present experimental evidence^45^, and (iii) possible interactions between chromate species and BAC functional groups^46^. Therefore, the large negative Δ*H* may indicate that adsorption is governed by strong adsorbate–adsorbent interactions rather than simple physisorption alone. Nevertheless, the magnitude of Δ*H* should be interpreted with caution because thermodynamic parameters derived from adsorption equilibrium data provide indirect evidence and do not identify the underlying interaction mechanism. However, the specific nature of these interactions cannot be conclusively determined from the present data and requires further spectroscopic investigation. Similar high values have been reported in the literature for Cr(VI) removal by modified adsorbents involving multiple^45,47^. The large negative Δ*S* (− 409.68 ± 9.5 J mol⁻¹ K⁻¹) signifies a substantial drop in randomness at the solid–solution interface during adsorption, which is consistent with the immobilization of Cr(VI) oxyanions onto organized adsorption sites within the BAC-modified interlayer. Such ordering may result from restricted mobility of both the adsorbed chromate species and the surrounding water molecules during adsorption. Nevertheless, the unusually large magnitude of Δ*S* should be interpreted cautiously because adsorption-derived thermodynamic parameters are sensitive to the method used for estimating the equilibrium constant and may also reflect the combined influence of several simultaneous adsorption phenomena rather than a single elementary adsorption mechanism. The magnitude of Δ*H* and Δ*G* tabulated in Table 4 is compatible with the occurrence of relatively strong adsorbate–adsorbent interactions, particularly electrostatic attraction between Cr(VI) oxyanions and positively charged BAC-modified surfaces. Overall, the thermodynamic parameters support favorable adsorption on BAC-modified bentonite; however, they should not be regarded as direct evidence of any specific adsorption mechanism. Confirmation of the nature of the adsorbate–adsorbent interactions will require advanced spectroscopic techniques such as XPS, XANES, or related surface-sensitive analyses^38,46^.

**Table 4:** Thermodynamic data for Cr (VI) sorption on NaB and OMB sorbents at various temperatures.

Sorbent	Temperature (K)	ΔG (kJ mol⁻¹)	ΔH (k J/mole)	ΔS (J mol⁻¹ K⁻¹)
NaB	303	6.64 ± 0.21	19.91 ± 0.72	44.23 ± 1.15
	313	5.79 ± 0.18		
	323	5.77 ± 0.17		
OMB	303	−14.27 ± 0.43	−137.65 ± 4.8	−409.68 ± 9.5
	313	−7.82 ± 0.26		
	323	−6.18 ± 0.22		

### Suggested mechanism, mechanistic considerations for the adsorption strategy, and comparison with alternative sorbents

The improved performance of BAC-modified bentonite can be attributed to the dual functionality of BAC molecules, which introduce quaternary ammonium groups that increase electrostatic attraction toward negatively charged chromate species (CrO₄²⁻/HCrO₄⁻), while also improving surface organophilicity, although the remarkable reduction in BET surface area implying that the adsorption process is predominantly controlled by surface charge characteristics and specific electrostatic interactions rather than by physical surface area alone. Compared to conventional surfactants, BAC’s molecular structure may facilitate a more accessible arrangement of active sites, contributing to improved adsorption efficiency at lower dosages. The adsorption stages followed pseudo-second-order kinetics, while intraparticle diffusion contributes but is not rate-limiting. Thermodynamic parameters confirmed a spontaneous and exothermic process for OMB, in contrast to the weak and non-spontaneous adsorption observed for raw NaB.

**Fig. 11:**
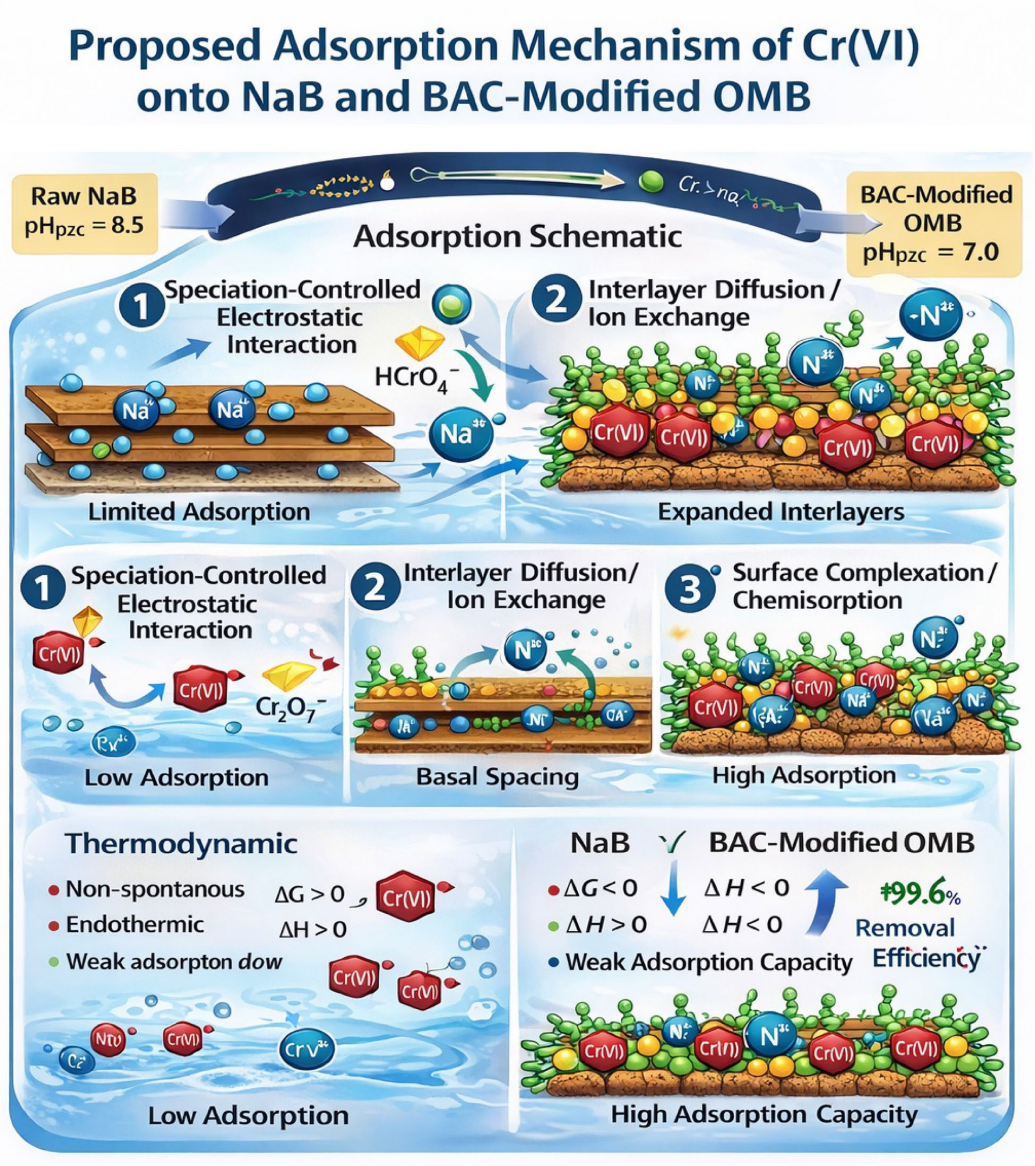
Suggested adsorption mechanism for Cr(VI) elimination onto NaB and OMB surfaces, illustrating the proposed adsorption pathways responsible for enhanced Cr(VI) uptake by OMB sorbent.

This enhanced Cr(VI) adsorption on OMB can be attributed to (i) increased interlayer spacing due to BAC intercalation, (ii) introduction of positively charged quaternary ammonium groups that electrostatically attract Cr(VI) oxyanions, and (iii) possible additional interactions between chromate species and BAC functional groups^22,24,27,28^. This modification changed the surface from predominantly cation-exchange to anion-affinitive, making OMB suitable for Cr(VI) removal at low pH, as noticed from the findings^27,28^. It should be emphasized that the proposed adsorption mechanism is inferred from adsorption behavior, characterization results, and thermodynamic analysis. Direct evidence for specific adsorption pathways was not obtained in the present study, and therefore the proposed mechanism should be regarded as tentative pending confirmation by advanced spectroscopic techniques such as XPS or XANES. This proposed mechanism is presented in Fig. 11. To further contextualize the performance of the proposed system, a quantitative comparison with previously reported organo-modified clays was presented in Table 5^48–53^,. Although a direct experimental comparison with other surfactants, such as CTAB and HDTMA, was not made in the present study, the literature-based evaluation showed that BAC-modified bentonite revealed comparable or improved Cr(VI) removal efficiency, particularly at lower adsorbent dosage. This better performance can be ascribed to several factors. First, BAC introduced positively charged quaternary ammonium groups that improved the electrostatic attraction toward negatively charged chromate species under acidic conditions. Second, XRD analysis confirmed expansion of the interlayer spacing after BAC-modification, which probably improved the accessibility of the adsorption sites. Third, the adsorption process exhibited favorable pseudo-second-order kinetics and Langmuir behavior, suggesting efficient monolayer adsorption on energetically favorable sites.

**Table 5:** Comparison of adsorption performance and operating conditions of BAC-modified bentonite with previously reported organo-clay adsorbents for Cr(VI) removal.

Adsorbent System	Modifier Type	Removal Efficiency (%)	Adsorbent Dose	Optimal pH	Numerical adsorption capacity (mg g⁻¹)	Key Mechanism	Reference
Bentonite (natural)	None	Low–moderate (< 50%)	Moderate–high	2–4	13–27	Limited anion adsorption	^48^
Bentonite (organoclay)	HDTMA	~ 90–98%	Moderate–high	~ 2–4	250	Electrostatic attraction via quaternary ammonium groups	^49^
Montmorillonite	HDTMA (micelle-clay)	~ 100% (under optimized conditions)	High	Wide pH range	9.43	Micelle-enhanced adsorption	^50^
Organobentonite/alginate beads	HDTMA	~ 55–66%	Moderate	~ 3–4	0.519	Ion exchange + electrostatic interaction	^51^
Modified bentonite	HDTMA/CPC	High (varies with surfactant type)	Moderate	Acidic	45.5–46	Surfactant-dependent adsorption enhancement	^52^
Zeolite (organoclay)	HDTMA	Moderate–high (capacity-driven)	Moderate	~ 4	0.068 − 0.0093	Surface charge reversal → anion adsorption	^53^
This work	BAC (benzalkonium chloride)	99.6%	Low	Optimized acidic	2.27	Enhanced electrostatic interaction + improved site accessibility	Present study

Also and compared with other low-cost adsorbents such as agricultural residues, biochars, and biosorbents (e.g., mango kernel powder, pomegranate peel, rice straw biochar, banana peel, palm oil fuel ash, lignin, and various clays and composites)^12,13,37,38,42,43^, OMB exhibited competitive or superior Cr(VI) adsorption capacity under similar acidic conditions. The facile preparation, abundance of bentonite, and relatively low cost of BAC made this modified material promising for practical wastewater treatment, especially in regions where bentonite deposits are available.

### Regeneration and reusability of OMB sorbent

To evaluate the practical applicability of OMB, its regeneration and reuse potential were performed based on the stability of the organoclay structure and the proposed adsorption mechanism. After Cr(VI) adsorption, desorption was achieved using a 0.1 M NaOH solution, which promotes the release of adsorbed chromate species through electrostatic repulsion and ion exchange under alkaline conditions. The other related optimum experimental conditions, i.e.,

(150% BAC-modified bentonite) OMB adsorbent, 10 mg L⁻¹ initial Cr(VI) concentration, 4 g L⁻¹ adsorbent dose, 200 rpm shaking speed, 70 min contact time, and 30 °C (303 K) temperature, were maintained throughout the regeneration experiments. After each cycle, the regenerated adsorbent was subsequently washed with distilled water until neutral pH and reused in consecutive adsorption cycles.

**Fig. 12:**
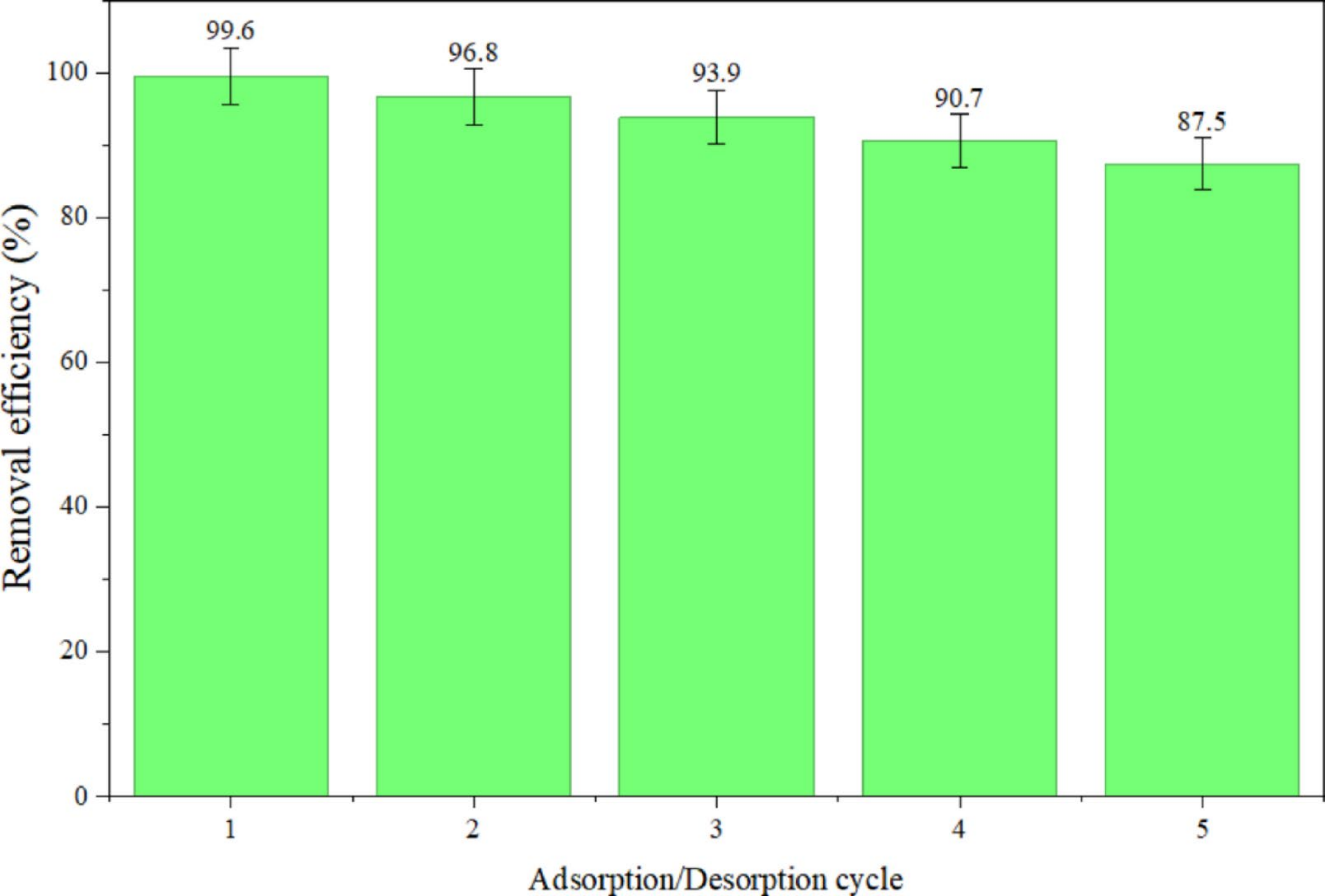
Representation of OMB sorbent dataset reusability for five adsorption/desorption cycles related to the removal of Cr (VI) species. Error bars represent the standard deviation (SD) of triplicate measurements.

Based on the structural stability indicated by FTIR and XRD analyses, as well as the preservation of interlayer organization after BAC modification, OMB retained high adsorption efficiency over five consecutive cycles. The Cr(VI) removal efficiency decreased gradually from 99.6% in the first cycle to 87.5% in the fifth cycle, corresponding to an overall loss of 12.1%. Similarly, the adsorption capacity decreased from 2.49 mg g⁻¹ in the first cycle to 2.18 mg g⁻¹after these five cycles. These reported values represent the mean of triplicate measurements, and the associated standard deviations are represented as error bars in Fig. 12. This decline in adsorption performance may be attributed to the partial occupation of active sites and a possible minor loss of surfactant during regeneration, thereby demonstrating the satisfactory structural stability and reusability of the BAC-modified bentonite under the investigated regeneration conditions. Although direct BAC leaching measurements were not performed, the retained adsorption efficiency after repeated regeneration cycles, together with the structural stability observed in FTIR and XRD analyses, suggests that extensive surfactant loss was unlikely under the investigated conditions. Nevertheless, direct quantification of BAC leaching should be considered in future studies.

The proposed regeneration behavior is consistent with previously reported organobentonite systems, where quaternary ammonium-modified clays retained substantial adsorption efficiency after multiple adsorption–desorption cycles^49^.

### Application to real industrial wastewater (Case study)

To evaluate the applicability of the prepared OMB under practical conditions, an industrial wastewater sample was investigated as a case study using the optimum adsorption conditions established in this work. Chromium-containing wastewater was collected from an electroplating facility located in the industrial zone of Alexandria Governorate, Egypt. Preliminary analysis confirmed the presence of chromium together with other dissolved species commonly found in electroplating effluents, including Na⁺, Ca²⁺, Cl⁻, and SO₄²⁻. However, the present investigation focused primarily on the Cr(VI) removal performance.

The initial chromium concentration in the wastewater sample was approximately 40.01 mg L⁻¹. The sample was treated using OMB under optimized adsorption conditions and agitated for 70 min at 200 rpm. After adsorption and filtration, the residual chromium concentration decreased to approximately 3 mg L⁻¹, corresponding to a removal efficiency of 92.5 ± 0.6% and an adsorption capacity of 9.26 ± 0.14 mg g⁻¹.

The successful removal of chromium from the industrial wastewater sample demonstrates that OMB maintained a high adsorption performance even in the presence of competing dissolved species. The observed removal efficiency was slightly lower than that obtained under idealized laboratory conditions, which may be attributed to competition between chromate species and coexisting ions for adsorption sites. In particular, sulfate and chloride ions may compete with chromate ions through electrostatic interactions, while dissolved cations may influence the electrical double layer surrounding the adsorbent surface. Nevertheless, the high chromium removal efficiency indicates that the BAC-modified bentonite retained a strong affinity for chromate species despite the complexity of the wastewater matrix.

This real wastewater experiment was intended as a preliminary proof-of-concept evaluation of the prepared OMB adsorbent’s suitability under realistic conditions, rather than as a comprehensive wastewater characterization study. Accordingly, physicochemical parameters such as chemical oxygen demand (COD), total dissolved solids (TDS), electrical conductivity, and the complete ionic composition of the wastewater were not measured. These factors may govern adsorption behavior through competitive interactions and changes in solution chemistry; therefore, their incorporation would provide a more comprehensive assessment of the proposed adsorbent’s practical performance. Nevertheless, the observed Cr(VI) removal efficiency of 92.5 ± 0.6% demonstrates that the BAC-modified bentonite retained high adsorption capability in a complex industrial wastewater matrix. Future work will include comprehensive wastewater characterization and a systematic investigation of the influence of competing inorganic ions and organic constituents under realistic operating conditions.

In sum, the developed OMB system offers several practical advantages, including high adsorption efficiency, low-cost raw materials, simple preparation methodology, applicability to real industrial wastewater, and encouraging regeneration potential. However, optimum adsorption was achieved under an acidic environment (pH = 2), which may increase operational costs associated with pH adjustment during large-scale application. Accordingly, further optimization under near-neutral conditions remains an important objective for future research.

## Conclusion

Benzalkonium-chloride-modified Egyptian sodium bentonite (OMB) was successfully synthesized and used for the efficient sequestration of Cr(VI) from aqueous phases. FTIR, XRD, BET, and *pH*_pzc_ analyses proved effective intercalation of BAC into the clay structure, expansion of the basal spacing, and modification of surface properties. Under optimized conditions (150% BAC dose, pH 2, 4 g L^− 1^ dose of clay sorbent, room temperature (30 °C), 200 rpm shaking speed, 70 min contact time), OMB achieved ≈ 99.6 ± 0.4% Cr(VI) removal from dilute solutions. Although the BAC modification markedly reduced BET surface area due to partial pore occupation and surfactant coverage, the inclusion of positively charged quaternary ammonium groups and expansion of the clay interlayer spacing significantly enhanced Cr(VI) adsorption through favorable electrostatic interactions and improved accessibility of active adsorption sites. In contrast, unmodified NaB removed only 6.6 ± 0.06% of Cr ions. Adsorption kinetics followed the pseudo-second-order model, indicating that the adsorption rate was well described by the model; however, this alone does not conclusively establish a chemisorption mechanism, while equilibrium data were best expressed by the Langmuir isotherm, suggesting monolayer adsorption at homogeneous sites. Thermodynamic analysis suggested that Cr(VI) adsorption on OMB was thermodynamically favorable, spontaneous, and exothermic under the investigated experimental conditions. Furthermore, the adsorption mechanism and structural stability of OMB suggested promising regeneration and reusability potential. The study found that BAC-modified bentonite is a cost-effective, abundant, and effective sorbent for Cr(VI) removal and has strong potential for application in industrial wastewater treatment. In general, the synergistic OMB system exhibited high removal performance, low-cost preparation, and promising regeneration capability. However, further optimization under less acidic conditions is necessary before a large-scale industrial application. Future investigations may focus on evaluating the performance of the developed OMB sorbent in continuous-flow adsorption processes. Additional studies involving advanced surface characterization techniques such as SEM/FESEM and TEM, together with surface-sensitive spectroscopic analyses (e.g., XPS, XANES, or related techniques), detailed diffusion modeling, and competitive ion adsorption systems, are required to verify the proposed the adsorption mechanism and identify the nature of adsorbate–adsorbent interactions, the spatial distribution of BAC, possible multilayer organization, the relative contribution of external and interlayer adsorption sites, and transport behavior. Furthermore, the large-scale applicability of the modified bentonite system should be explored to support its practical applicability in wastewater treatment applications.

## Data Availability

All data generated or analyzed during this study are included in this published article.
